# Isolation and Characterization of Biosurfactant-Producing Bacteria From Oil Well Batteries With Antimicrobial Activities Against Food-Borne and Plant Pathogens

**DOI:** 10.3389/fmicb.2020.00064

**Published:** 2020-02-27

**Authors:** Mamta Rani, Joel T. Weadge, Suha Jabaji

**Affiliations:** ^1^Department of Plant Science, Faculty of Agricultural and Environmental Sciences, McGill University, Montreal, QC, Canada; ^2^Department of Biology, Wilfrid Laurier University, Waterloo, ON, Canada

**Keywords:** biosurfactant producing bacteria, oil wells, human pathogens, *Salmonella*, *Xanthomonas campestris*, biological control, plants

## Abstract

Microbial biosurfactants, produced by fungi, yeast, and bacteria, are surface-active compounds with emulsifying properties that have a number of known activities, including the solubilization of microbial biofilms. In an on-going survey to uncover new or enhanced antimicrobial metabolite-producing microbes from harsh environments, such as oil-rich niches, 123 bacterial strains were isolated from three oil batteries in the region of Chauvin, Alberta, and characterized by 16S rRNA gene sequencing. Based on their nucleotide sequences, the strains are associated with 3 phyla (Actinobacteria, Proteobacteria and Firmicutes), as well as 17 other discrete genera that shared high homology with known sequences, with the majority of these strains identified to the species level. The most prevalent strains associated with the three oil wells belonged to the *Bacillus* genus. Thirty-four of the 123 strains were identified as biosurfactant-producers, among which *Bacillus methylotrophicus* strain OB9 exhibited the highest biosurfactant activity based on multiple screening methods and a comparative analysis with the commercially available biosurfactant, Tween 20. *B. methylotrophicus* OB9 was selected for further antimicrobial analysis and addition of live cultures of *B. methylotrophicus* OB9 (or partially purified biosurfactant fractions thereof) were highly effective on biofilm disruption in agar diffusion assays against several Gram-negative food-borne bacteria and plant pathogens. Upon co-culturing with *B. methylotrophicus* OB9, the number of either *Salmonella enterica* subsp. *enterica* Newport SL1 or *Xanthomonas campestris* B07.007 cells significantly decreased after 6 h and were not retrieved from co-cultures following 12 h exposure. These results also translated to studies on plants, where bacterized tomato seedlings with OB9 significantly protected the tomato leaves from *Salmonella enterica* Newport SL1 contamination, as evidenced by a 40% reduction of log_10_ CFU of *Salmonella*/mg leaf tissue compared to non-bacterized tomato leaves. When *B. methylotrophicus* 0B9 was used for bacterized lettuce, the growth of *X. campestris* B07.007, the causal agent of bacterial leaf spot of lettuce, was completely inhibited. While limited, these studies are noteworthy as they demonstrate the inhibition spectrum of *B. methylotrophicus* 0B9 against both human and plant pathogens; thereby making this bacterium attractive for agricultural and food safety applications in a climate where microbial-biofilm persistence is an increasing problem.

## Introduction

Organic pollutants, such as crude petroleum, are commonly assumed to be a harsh habitat/medium for microbes to thrive upon. However, in spite of its high toxicity and hydrophobicity, mounting evidence has revealed the presence of live microbes (mainly bacteria) in crude oil. A wide distribution of microbial diversity, including *Pseudomonas, Bacillus*, *Streptomyces* and *Stenotrophomonas* species, were associated with oil wells (Yoshida et al., 2005; Cai et al., 2015). These organisms have the potential to degrade numerous hydrocarbons for use as carbon sources and encapsulate heavy metals and/or hydrocarbons through the production of active surfactants (Varjani and Gnansounou, 2017).

Biosurfactants are surface-active compounds with emulsifying activities. These compounds are described as amphiphilic, containing both hydrophilic and hydrophobic ends, that allows them to interact at the interface between aqueous and non-aqueous systems (Marchant and Banat, 2012). There are six classes of biosurfactants: glycolipids, lipopeptides or lipoproteins, neutral lipids, phospholipids, substituted fatty acids and lipopolysaccharides. Based on literature, the class of biosurfactant the genus *Bacillus* produces is lipopeptide (Zhao et al., 2017). Most of the work on biosurfactant application is focused on the microbially enhanced oil recovery (MEOR) technique, which relies on the use of microbial surfactants or killed bacterial cultures to liberate crude oil from binding surfaces, such as rocks and crevices (Perfumo et al., 2010), and to bioremediate of pollutants (Mulligan, 2005; Thavasi, 2011). However, in recent years microbial biosurfactants have gained renewed commercial interest because they have several advantages (e.g., low toxicity to the environment, eco-friendly, biodegradability and acceptability) over their synthetic counterparts that makes the biosurfactants amenable for application in the fields of agriculture, the food industry and therapeutics (Nitschke and Costa, 2007; Sachdev and Cameotra, 2013; Mani et al., 2016).

A large number of reports have described a variety of biosurfactant producers originating from aqueous environments and polluted/unpolluted soils with crude oil (Sachdev and Cameotra, 2013). Many rhizosphere and plant-associated microbes are biosurfactant producers, strongly suggesting their potential role in plant-microbe interactions and in broader agriculture (Marchut-Mikolajczyk et al., 2018) regarding combating plant diseases and foodborne pathogens (Moore et al., 2013). For example, several biosurfactant-producing bacterial strains of *Pseudomonas* and *Bacillus* species were effective in controlling seed rots, damping off, leaf spots, blight and wilts of several crops (Debode et al., 2007; D’aes et al., 2010; Sachdev and Cameotra, 2013). Other strains were effective against food-borne pathogens, including *Salmonella* species and *Escherichia coli* (Díaz De Rienzo et al., 2015; Hsu and Micallef, 2017). Therefore, isolation and characterization of bacteria with promising biosurfactant and/or antimicrobial properties from unexplored environmental samples, such as crude oil, may have applications in plant disease suppression and in the reduction of food–borne pathogens, thus warranting further research.

Some of the leading causes of food-borne illness are *Salmonella* and *E. coli* O157:H7 (Lynch et al., 2009). The Salmonellosis and *E. coli* O157:H7 outbreaks associated with fresh produce have been attributed most frequently to consumption of tomatoes, cantaloupes and leafy greens (Murray et al., 2017). Edible plants that become contaminated during agricultural practices can transmit the pathogen to consumers leading possibly to Salmonellosis outbreaks (Snyder et al., 2019). Mounting evidence indicates that food-borne pathogens not only contaminate plant surfaces, but are also able to internalize into the tissues of fresh produce during the growing and distribution processes (Deering et al., 2012). Thus, reducing *Salmonella enterica* association with plants during crop production could reduce risks of fresh produce-borne Salmonellosis.

In addition to being a vector of food-borne disease, fresh vegetables are of economic concern with respect to pathogens that directly result in destruction of the plant and its resultant yield. One of the most devastating diseases of lettuce is bacterial leaf spot (BLS) disease, caused by *Xanthomonas campestris*. Water soaked lesions appear on the margins of the leaves, reducing the quality and affecting the market value of lettuce (Nicolas et al., 2018). In Quebec (Canada), severe outbreaks of BLS have led to devastating economic losses (i.e., 100% of the lettuce in the field is ruined). Recently, biological control of plant pathogens using antagonistic bacteria has presented itself as a promising strategy for plant protection (Heydari and Pessarakli, 2010). Lactic acid bacteria (LAB) and antagonistic strains of *Bacillus subtilis* were reported to be effective against different pathovars of *X. campestris* (Wulff et al., 2002; Trias et al., 2008; Saleem et al., 2017). Thus, the discovery of additional antagonistic bacteria that have activity against *X. campestris* pv. Vitians, as well as other human/plant pathogens merits exploring.

We hypothesized that oil-dwelling bacterial strains can be a source of novel biosurfactant compounds with enhanced prophylactic abilities against human and plant pathogens. The objectives of this study were to (i) isolate and characterize bacterial strains from crude-oil samples, (ii) demonstrate the biosurfactant-producing potential of each of these strains using multiple screening methods, and (iii) as a proof-of-principal, identify the top biosurfactant-producer and assay it against a collection of human (including food-borne) and plant pathogens for highly antagonistic activity in co-culture and protective capabilities *in planta.*

## Materials and Methods

### Source of Crude Oil Samples and Organisms

Crude oil samples were obtained from the operating area of Talisman Energy Inc., which is located in Chauvin, Alberta (Supplementary Figure S1). We were provided with duplicate samples representing pooled samples from wells of each of the three batteries (battery 10–22, battery 5–27 and battery 10–17), using sterile 500 mL Schott bottles tightly sealed, kept on ice during transportation and stored at 4°C for DNA extraction (Table 1). The physical and chemical characteristics of the crude oil are listed in Table 1. Target organisms, consisting of a panel of 41 bacterial and fungal strains (listed in Supplementary Table S1), were tested in dual diffusion assays with biosurfactant-producing strains. *Salmonella enterica* subsp. *enterica* strains belonging to 17 different serovars, including top clinical serovars (e.g., Typhimurium, Heidelberg, Newport, Infantis, Thompson and Braenderup) and environmental samples, were classified according to the four *Salmonella* biofilm morphotypes displayed on congo red plates (Römling et al., 1998). Strains with the morphotypes: rdar (red dry and rough) are cellulose and curli positive, pdar (pink dry and rough) are cellulose positive, bdar (brown dry and rough) are curli positive and saw (smooth and white) are negative for both components.

**TABLE 1:** Physical and chemical properties of oil samples from oil batteries 10–22, 5–27, and 10–17.

Properties	Location		
Oil Battery	Chauvin, Alberta 10–22	Chauvin, Alberta 5–27	Chauvin, Alberta 10–27
Number of wells^a^	36	119	162
pH	7.4	7.4	7.4
Specific gravity	1.06 kg/m^3^	1.06 kg/m^3^	1.06 kg/m^3^
Water content	0.40%	0.29%	0.14%
Absolute density	955.1 kg/m^3^	917 kg/m^3^	946.7 kg/m^3^
API gravity	16.6 kg/m^3^	22.8 kg/m^3^	17.9 kg/m^3^
Sulfur	25.3 g/kg	24.7	28.7 g/kg
Total solids dissolved	81315 mg/lt	82900 mg/lt	86159 mg/lt
Viscosity	863 mPas	87.05 mPas	644.5 mPas

*^*a*^Recovery and isolation of microbes were pooled from duplicate oil samples provided from each battery.*

### Isolation of Oil-Dwelling Bacteria

Unless otherwise stated, manipulation of oil samples was performed under sterile conditions in a laminar flow hood in order to avoid any contamination. Crude oil samples (10 mL of each) were mixed with an equal volume of sterile distilled water. The emulsion was vortexed and then continuously agitated (2 × *g*) on a rotary shaker for 2 h, before the aqueous phase was recovered with a sterile pipette. This procedure was repeated five times, collecting a total of 50 mL of the aqueous phase for each sample from each well. Aliquots (100 μL) of the aqueous phase were plated on different microbiological culture media; potato dextrose agar (PDA), malt extract agar (MEA), nutrient agar (NA) and lysogeny broth agar (LBA) (BDH chemical Ltd., Mississauga, ON, Canada), and incubated at 28°C. Bacterial colonies that grew on culture media were passed through four rounds of single colony isolation by streaking them on the above-mentioned culture media to ensure purity of each strain prior to long-term storage. Pure bacterial cultures were stored in 20% (v/v) glycerol and kept at −80°C until further use.

### Molecular Identification of Bacteria Recovered From Oil

Bacterial strains were grown in LB broth for 18 h with agitation to obtain a final concentration of 10^8^–10^10^ colony forming units (CFU) mL^–1^. Cells were pelleted by centrifugation, and DNA was extracted using the DNeasy^®^ Blood & Tissue kit (Qiagen, Mississauga, ON, Canada) following the manufacturer’s instructions. DNA concentration and quality were confirmed spectrophotometrically with a NanoDrop ND1000 spectrophotometer (NanoDrop, Wilmington, DE, United States) and on a 1% (w/v) agarose gel, respectively. The 16S rRNA gene sequences were amplified using the ITS primer pair 27F/534R (5′-AGAGTTTGATCCTGGCTCAG-3′) and 534R (5′-ATTACCGCGGCTGCTGG-3′) according to previously published protocols (Gagne-Bourgue et al., 2013; Scott et al., 2018) to putatively identify 123 bacterial strains. PCR amplification was performed in a Bio-Rad T100 Thermal Cycler using the iProofTM High-Fidelity (HF) PCR kit (Bio-Rad, Ontario, Canada) using 40 ng of genomic DNA for a 50 μL reaction following previously published protocols (Gagne-Bourgue et al., 2013; Scott et al., 2018). Negative and positive controls were included concurrently with each reaction according to previous protocols (Scott et al., 2018). Amplification of PCR products was confirmed on a 1% (w/v) agarose gel. Lower molecular weight PCR products were cloned into the pDrive vector (Qiagen) following the manufacturer’s protocol. Purified plasmid DNA and PCR products were sequenced at Genome Quebec sequencing services (McGill University, Montreal, QC, Canada). Sequences were subjected to Blast search against the NCBI database using the algorithm megablast^1^ to confirm identity through sequence homology. The obtained sequences were submitted to NCBI GenBank.

### Enrichment of Biosurfactant Production by Oil-Dwelling Bacteria

For efficient degradation of complex hydrocarbon oil and the production of biosurfactants, 3.27 g/L of Bushnell and Haas (BHM) (BDH chemical Ltd) supplemented with 2% of each of crude oil (v/v), glucose (w/v) and molasses (v/v) as sole sources of carbon, adjusted to pH 7.0 and sterilized at 21 psi for 20 min was used. A 1 mL volume of bacterial cultures (grown at 22°C for 18–24 h with agitation in LB broth) with an OD_600_ between 0.6 and 1.0 was transferred to 100 mL of the carbon-amended BHM media. Inoculated media was incubated with continuous agitation (2 × *g*) at 30°C for 7 days and then the cell-free supernatant was collected by centrifugation (6500 × *g* at 4°C for 20 min). Removal of any residual oil and/or live bacterial cells present in the cell-free supernatant was accomplished by filtration using a 0.22 μm Millipore filter (Millipore Sigma, Ontario, Canada) and kept at 4°C until further use.

### Assays for Biosurfactant Production

Bacterial isolates originating from oil batteries (10–22, 5–27, and 10–17) were screened for biosurfactant production by applying the most commonly used assays in the literature; the oil-spreading test and drop-collapse assay (Youssef et al., 2004; Thavasi, 2011). Isolates were considered to have significant biosurfactant production if the clearing zone they produced was at least ≥ 1.0 cm in diameter in the oil spreading assay and ≥ 3.0 mm in the drop collapse assay (Youssef et al., 2004). In all assays unless otherwise stated, Triton X-100 (10 mg/mL) was used as the positive control, while sterile de-ionized water and un-inoculated hydrocarbon-amended BHM medium served as negative controls. All tests were replicated twice for each bacterial strain tested. Based on the above-mentioned criteria, the top biosurfactant producers were further screened using the CTAB agar method, emulsification assay, microplate assay, and hemolytic assay.

#### Drop Collapse Assay

The wells of a polystyrene 96 well micro-plate lid (Corning Incorporated, United States) were coated with 2 μL of crude oil and left to dry for 24 h at 22°C. Filtered cell-free supernatant (5 μL) was transferred to the center of the oil coated well. The results were recorded after 1–2 min and considered positive for biosurfactant production when the oil drop was flat. Those that gave rounded drops were scored negative, an indication of the absence of biosurfactant production (Jain et al., 1991).

#### Oil Spreading Assay

The oil-spreading assay was performed in polystyrene petri dishes (100 mm × 15 mm) containing 20 μL of crude oil that was carefully layered over 20 mL of distilled water. A drop (∼10 μL) of filtered supernatant was carefully pipetted onto the center of the oil layer. The diameter of the clear zone on the surface of the oil layer was measured and compared to the negative controls (Ibrahim et al., 2013).

#### CTAB Agar Assay

CTAB agar plates were prepared by adding 0.15 g of cetyltrimethylammonium bromide (CTAB; Sigma-Aldrich, Oakville, ON, Canada), 0.005 g Methylene blue (Sigma-Aldrich) and 12 g of agar to 1 L of distilled water, adjusted to pH 7 and sterilized (Tahzibi et al., 2004). Two holes (6.5 mm diameter) were made in the CTAB plates, and approximately 150 μL of filtered cell-free supernatant was loaded inside each hole. Plates were incubated at 37°C for 48 h. Cell free supernatant containing anionic surfactant produced blue halos around the wells in which they were placed. The diameter of the halo was measured and compared with positive and negative controls.

#### Emulsification Assay

A volume of 1 mL of the cell-free supernatant was added to 5 mL of 50 mM Tris buffer (pH 8.0) in a 30 mL screw-capped test tube. Crude oil was tested for emulsification activity. Crude oil (5 mg) was added to both layers and vortexed for 1 min and then the emulsion mixture was allowed to settle for 20 min. The optical density of the emulsified mixture was measured at 610 nm (Muthezhilan et al., 2014). A negative control consisted of only buffer solution and crude oil with Triton X-100 was used as the positive control.

#### Microplate Assay

An aliquot of 50 μL of filtered cell-free supernatant was placed in domed PCR caps (Ultident Scientific, St. Laurent, QC, Canada) that were oriented over a grid of 1 mm × 1 mm squares. The presence of biosurfactant was confirmed by the distortion of the grid image and were qualitatively compared to the positive and negative controls (Walter et al., 2010).

#### Hemolytic Activity

Blood agar plates were prepared by adding 5% (v/v) of sheep blood (Fisher scientific) to a sterilized mixture of NaCl (10 g), yeast (5 g), tryptone (10 g) and agar (15 g) in 1 L of distilled water (Phan et al., 2013). Approximately 150 μL of filtered cell free supernatant of each bacterial isolate was loaded into each well (6.5 mm in diameter) made by a cork borer in the blood agar plates and incubated at 30°C for 24–48 h. Biosurfactant biosynthesis was confirmed by hemolysis activity as indicated by the presence of clearing zones around the wells. The diameter of the lysis zone is scored as ‘–’no lysis, ‘ + ’ partial hemolysis, ‘ + + ’ moderate hemolysis, ‘ + + + ’ complete hemolysis.

### Antimicrobial Activity of Whole Cultures of *B. methylotrophicus* OB9

Isolate OB9 exhibited the highest activity for oil displacement and emulsification activity. This strain was identified as *B. methylotrophicus* based on sequencing data and hereby designated as *B. methylotrophicus* OB9 (Jeukens et al., 2015). This strain was further screened for its antimicrobial activity against a wide panel of clinical and environmental bacterial strains (Table 2) employing the Burkholder agar diffusion assay (Burkholder et al., 1944), and also against fungal phytopathogens using the dual confrontation assay (Gagne-Bourgue et al., 2013).

**TABLE 2:** Top biosurfactant-producing isolates of three oil wells 10–22, 5–27, 10–17.

Rank	Name	Strain ID	Oil spreading (cm)^a^	Drop collapse (mm)^b^
**Well 10–22**				
1	*Bacillus megaterium*	OB55	1.6 ± 0.1^c^	3.5 ± 0.0
2	*Streptomyces* sp.	OB45	1.6 ± 0.7	3.0 ± 0.0
3	*Streptomyces* sp.	OB44	2.0 ± 0.6	3.5 ± 0.0
4	*Bacillus amyloliquefaciens*	OB10	2.1 ± 0.2	3.5 ± 0.0
5	*Bacillus nealsonii*	OB51	2.6 ± 0.0	4.0 ± 0.0
6	*Microbacterium* sp.	OB14	2.3 ± 0.2	3.5 ± 0.0
7	*Bacillus methylotrophicus*	OB43	2.4 ± 0.7	3.5 ± 0.0
8	*Streptomyces yanglinensis*	OB41	2.6 ± 0.1	3.5 ± 0.0
9	*Bacillus amyloliquefaciens*	OB5	3.2 ± 0.3	3.8 ± 0.3
10	*Streptomyces* sp.	OB42-1	3.3 ± 0.1	3.5 ± 0.1
11	*Bacillus amyloliquefaciens*	OB6	3.6 ± 0.3	4.3 ± 0.3
12	*Bacillus methylotrophicus*	OB9	3.8 ± 0.1	4.3 ± 0.6
**Well 5–27**				
1	*Bacillus simplex*	E05	1.5 ± 0.0	3.5 ± 0.0
2	*Micrococcus luteus*	E47	1.5 ± 0.2	3.3 ± 0.1
3	*Rhodococcus* sp.	E51-2	1.6 ± 2.7	3.5 ± 0.0
4	*Bacillus methylotrophicus*	E62	1.7 ± 0.3	3.7 ± 0.2
5	*Pseudomonas* sp.	E14	2.2 ± 0.1	4.8 ± 0.3
6	*Bacillus subtilis*	E64	2.0 ± 0.2	3.8 ± 0.3
7	*Bacillus subtilis*	E68	2.6 ± 0.3	3.8 ± 0.0
8	*Arthrobacter* sp.	E45	2.9 ± 0.4	4.2 ± 0.3
**Well 10–17**				
1	*Bacillus methylotrophicus*	M16-3	2.2 ± 0.0	3.4 ± 0.1
2	*Bacillus subtilis*	M46-2	2.6 ± 0.1	3.3 ± 0.1
3	*Bacillus methylotrophicus*	M05-2	2.7 ± 0.2	3.4 ± 0.1
4	*Bacillus subtilis*	M43	2.9 ± 0.1	3.4 ± 0.2
5	*Bacillus amyloliquefaciens*	M36	2.9 ± 0.1	3.4 ± 0.2
6	*Bacillus amyloliquefaciens*	M27	3.0 ± 0.1	3.2 ± 0.1
7	*Bacillus subtilis*	M50	3.0 ± 0.1	3.4 ± 0.1
8	*Rhodococcus globerulus*	M20	3.1 ± 0.1	3.5 ± 0.0
9	*Staphylococcus saprophyticus*	M44	3.1 ± 0.0	3.2 ± 0.1
10	*Bacillus amyloliquefaciens*	M05	3.2 ± 0.2	3.4 ± 0.1
11	*Bacillus amyloliquefaciens*	M30	3.4 ± 0.1	3.4 ± 0.1
12	*Bacillus methylotrophicus*	M09	3.4 ± 0.0	3.4 ± 0.2
13	*Bacillus amyloliquefaciens*	M21	3.4 ± 0.1	3.2 ± 0.1
14	*Bacillus subtilis*	M42	3.5 ± 0.0	3.2 ± 0.1

*^*a*^For oil spreading assays a clearing zone of ≥1.5 cm is considered positive. ^*b*^For drop collapse assay a value above ≥ 3 mm is considered positive. ^*c*^Values represent the mean of three replicates ± standard error of the mean (S.E.).*

#### Agar Diffusion Assay

All target bacterial strains and *B. methylotrophicus* OB9 were grown in LB broth at 27°C for 16-18 h with constant shaking to achieve mid-log phase, with a cell density of 10^6^ CFU mL^–1^ as assessed based on standard curves relating optical density at 600 nm (OD_600_) to plate counts on LBA plates. Cells were pelleted by centrifugation (6500 × *g* at 4°C for 20 min) and suspended in sterile ddH_2_O. A total volume of 30 μL of each bacterial suspension was mixed gently with 2.5 mL of molten half-strength LB agar and poured into culture plates. An aliquot (10 μL) of *B. methylotrophicus* OB9 was spotted in the center of the bacterial lawn and plates were incubated at 24°C. Zones of bacterial growth-inhibition subjacent to the spotted *B. methylotrophicus* OB9 inoculum were recorded after 24–48 h and were found to vary in diameter between 3 and 6 mm depending on the potency of *B. methylotrophicus* OB9 antimicrobials that diffused into the agar. Individual trials were performed in triplicate and the entire assay was repeated twice to confirm the findings.

#### Dual Confrontation Assay

Confrontation assay plates were used to screen *B. methylotrophicus* OB9 for its ability to inhibit radial growth of *Botrytis cinerea*, and *Rhizoctonia solani* isolates according to the modified method of Gagné-Bourgue (Gagne-Bourgue et al., 2013). PDA plates were inoculated in the center with a 5 mm diameter mycelial plug taken from the edge of an actively growing fungal colony. A 5 μL volume of *B. methylotrophicus* OB9 (10^6^ CFU mL^–1^) was deposited 25 mm on either side of the fungal colony. Triplicate plate assays were performed for each target fungal organism and radial growth inhibition of the fungus was measured 5 days post confrontation.

### Purification and Fractionation of Biosurfactants of *B. methylotrophicus* OB9 by Thin Layer Chromatography (TLC)

The biosurfactant-producing *B. methylotrophicus* OB9 was cultured in 2 L of BHM broth supplemented with 2% (w/v) glucose, 2% (v/v) molasses and 2% (v/v) oil for 7 days with agitation (2 × *g)* at 30°C. Bacterial cells were removed by centrifugation (5000 × *g* at 4°C) and the cell–free supernatant (CFS) was acidified to pH 2.0 with 2N HCL at 4°C for 16 h, resulting in a precipitate that was subsequently collected by centrifugation (5000 × *g* for 10 min at 4°C). The acid precipitate fraction (APF) was dissolved in water (100 mg/mL) and adjusted to pH 7.0 using 1N NaOH. The CFS sample and clean BHM culture medium were concentrated by freeze-drying for 48 h. All samples were stored at −80°C until further use.

Separation of CFS and APF fractions was performed by thin layer chromatography (TLC). Acid-cleaned glass plates (20 × 20 cm) were coated with a thin layer of silica emulsion [160 g of silica with 15% (w/v) calcium sulfate and with fluorescent indicator GF254 dissolved in 500 mL of double distilled water] purchased from Sigma-Aldrich and dried for 16–18 h at 22°C. Activation of silica plates was done by heating the TLC plates at 120°C for 30 min prior to use. An amount of 100 mg of each of CFS and APF were dissolved in 400 μL of chloroform: methanol (2:1, v/v) and separated on activated TLC plates with chloroform: methanol: water (70:26:4) as the developing solvent system. Plates were observed under UV (λ = 254 nm) and the retention factor (Rf) values were calculated as distance traveled by the samples over the distance traveled by solvent. Only APF separated fractions were used for bioactivity assays. Bands corresponding to the desired Rf values were scraped and collected for extraction. Bands of the same Rf value from different plates were pooled together and extracted twice with chloroform: methanol (v/v/; 2:1). The collected fractions were vacuum-concentrated for 4–6 h at −60°C using a Speed Vac v78100 (Labconco Corp, Kansas City, MO, United States), re-dissolved in 50 μL of methanol and tested against target bacteria and fungi, as well as for biosurfactant activity. Methanol (50 μL) was used as a negative control.

### Biosurfactant and Antimicrobial Activities of TLC Fractions

The biosurfactant activity of the fractions was tested using the oil-spreading and the drop collapse methods, as described above. Fractions were also tested for their antimicrobial activities against a number of bacteria and fungi using the Burkholder diffusion plate method as noted above. Briefly, 10 μL of the dissolved fractions were spotted in the middle of the plates. Positive controls consisted of 10 μL of bleach and of each of the following organic acids: 1% (v/v) of acetic acid, formic acid, citric acid and lactic acid. All plates were incubated at 24°C and a clearing zone of 3 mm or greater after 24 h of incubation was considered positive for inhibition of growth. Individual trials were performed in triplicate and the entire assay was repeated twice to confirm the findings.

### Interaction of *B. methylotrophicus* OB9 With *S. enterica* Newport SL1 and *X. campestris* B07.007 in Liquid Co-culture

This method was performed to study how *B. methylotrophicus* OB9 directly interacts with target strains of *Salmonella enterica* Newport SL1 and *X. campestris* B07.007. Each bacterium was grown in LB broth until a cell density of 10^6^ CFU mL^–1^ was achieved, as assessed based on standard curves relating optical density at 600 nm (OD_600_) to plate counts on LBA plates. An equal volume of *B. methylotrophicus* OB9 culture and target organisms (*Salmonella* or *Xanthomonas*) was added to fresh LB liquid culture amended with 3% (w/v) yeast extract. Pure cultures of *Salmonella enteric* Newport SL1, *X. campestris* and *B. methylotrophicus* OB9 grown in amended LB broth served as positive controls. Liquid co-cultures and controls were incubated with agitation (2 × *g*) at 28°C. To avoid changes in growth during co-culturing due to pH variation or nutrient limitations, every 10 h liquid co-cultures and controls were subjected to centrifugation (15 min at 5000 × *g*) and bacterial pellets were suspended in fresh amended LB medium. Serial dilutions (10^–3^ to 10^–5^) from control and liquid co-cultures were plated on LBA after 3, 6, 12, 24, and 72 h of incubation, estimated as log_10_ CFU mL^–1^ and compared to the control treatments. There were three replicates per target organism and treatment. Individual trials were also repeated at least twice.

### Bacterization and Internalization of *B. methylotrophicus* OB9 in Tissue Cultured Tomato and Lettuce Seedlings

#### Vegetable Cultivars

Organic Tomato (cv. Beefsteak) and Cos lettuce (cv. Parris Island) seeds (Vesey seed Ltd, York, PE, Canada) were surface sterilized using 70% (v/v) ethanol and 1.3% (v/v) sodium hypochlorite in a stepwise procedure according to Scott et al. (2018). The seeds were transferred to tissue culture tubes (25 mm × 150 mm; VWR, Monroeville, PA, United States) containing 10 mL of Murashige and Skoog (MS) medium supplemented with 3% (w/v) sucrose (Gagné-Bourque et al., 2015). Tissue culture tubes were incubated under 200 μmol m^–2^ s^–1^ white fluorescent light, 18/6 h photoperiod, and 24°C day/night temperature for 2 weeks.

#### Preparation of *B. methylotrophicus* OB9 Inoculum

Inoculum of *B. methylotrophicus* OB9 was prepared by transferring a single colony to 100 mL of LB broth and incubating with agitation (2 × *g*) at 28°C. Bacterial cells were harvested by centrifugation (4500 × *g* for 15 min at 4°C), washed with 10 mM phosphate buffer (PBS) containing 0.8% (w/v) NaCl, pH 6.5 (phosphate buffered saline or PBS) and then suspended in the same buffer following another round of centrifugation (Pillay and Nowak, 1997). The density of the *B. methylotrophicus* OB9 suspension was adjusted to 10^6^ CFU mL^–1^ as described above.

#### Bacterization of Plant Seedlings With *B. methylotrophicus* OB9

A 500 μL volume of *B. methylotrophicus* OB9 in PBS buffer was carefully dispensed on the surface of the MS media and close to the roots of 2-week-old tomato and lettuce seedlings. Non-bacterized seedlings received 500 μL of PBS only and served as negative controls. Inoculated and control seedlings were returned to the environmental growth chamber and grown for 2 more weeks. The experimental unit was an individual plant contained in a test tube (a replicate) and five replicates per treatment were evaluated in two separate experimental trials. Bacterial populations were transformed to obtain homogeneity of variances and expressed as log_10_ (CFU per leaf fresh weight).

#### Colonization, Internalization and Retrieval of *B. methylotrophicus* OB9 From Plant Tissue

Recovery and colonization of plant tissues by *B. methylotrophicus* OB9 was confirmed by culture-dependent (cell count) and culture-independent (PCR assay) methods. Cell counts (CFU mL^–1^) were estimated 2 weeks post inoculation (wpi). Root and leaf tissues (100 mg of each) of tomato and lettuce seedlings were surface sterilized as described above to ensure that only *B. methylotrophicus* OB9 that had internally colonized plant tissues remained. The efficiency of the sterilization procedure was tested using the imprint method and absence of growth on the imprinted culture medium indicated that the surface sterilization procedure was effective. Surface sterilized tissues (100 mg) were ground in sterile distilled water, serially diluted (10^–1^ to 10^–7^) and spread on LBA plates to assess the presence of *B. methylotrophicus* OB9. The presence of *B. methylotrophicus* OB9 inside plant tissues was also confirmed by PCR amplification. Briefly, bacterized Plant DNA (200 mg) was reduced to powder in liquid nitrogen and genomic DNA was extracted using the CTAB extraction method (Porebski et al., 1997; Huang et al., 2011) that targeted the bacterial DNA in the plants. Genomic DNA from pure colonies of *B. methylotrophicus* OB9 was extracted and PCR was performed (Huang et al., 2011). The presence of *B. methylotrophicus* OB9 DNA in plant tissue was detected using specific primers [5′-CAAGTGCCGTTCAAATAG-3′ (Forward) and 5′CTCTAGGATTGTCAGAGG-3′ (Reverse)] in 25 μL PCR reactions using 20 ng of DNA. The amplification and PCR conditions are described in details in Gagné-Bourque et al. (2015).

### Protection of Tomato and Lettuce Leaves by *B. methylotrophicus* OB9 From *Salmonella* and *Xanthomonas*

#### Preparation of *Salmonella* and *Xanthomonas* for Leaf Inoculation

*Salmonella enterica* Newport SL1 (isolated from human gut) and *X. campestris* B07.007 (isolated from lettuce) were prepared by separately transferring a single colony of each strain into 100 mL of LB broth and incubating with agitation (2 × *g*) at 28°C. Bacterial cells were harvested, and the density of each culture was adjusted to 10^6^ cells mL^–1^ following the same procedures described for *B. methylotrophicus* OB9 cells above. Two separate experiments were set up to study the effect of *B. methylotrophicus* OB9 on growth of *Salmonella* and *Xanthomonas* on plants.

#### Experiment 1. Impact of Bacterized Plants With *B. methylotrophicus* OB9 on the Growth of *Salmonella* and *Xanthomonas*

Tomato and lettuce leaves of *B. methylotrophicus* OB9 bacterized plants were detached and placed separately into petri plates lined with sterile filter paper that was moistened with sterile distilled water. An aliquot (100 μL) of each of *Salmonella enterica* Newport SL1 and *X*. *campestris* B07.007 inoculum (10^6^ cells mL^–1^) was spread with a disposable spreader (Fischer Scientific Ltd) onto the surface of a tomato or lettuce leaf (*S. enterica* Newport SL1 and *X*. *campestris* B07.007, respectively). Non-bacterized leaves of tomato and lettuce were inoculated in the same manner and served as negative controls. All plates were sealed with parafilm and incubated at 22°C. Recovery of bacterial strains was determined by cell count after 72 h. Leaf tissue (100 mg) from infected and bacterized or non-bacterized plants was macerated in distilled water and serially diluted (10^–1^ to 10^–7^) and aliquots from each dilution were plated on LBA plates and bacterial cell counts were estimated and reported as log_10_ mL^–1^. Detection of *Salmonella* on tomato leaves was confirmed by PCR with *Salmonella* specific primers ST11 (5′-GC CAA CCA TTG CTA AAT TGG CGC A-3′) and ST15 (5′-GGT AGA AAT TCC CAG CGG GTA CTG-3′) using an annealing temperature of 57°C with PCR conditions described previously (Soumet et al., 1999). Detection of *X. campestris* on lettuce leaves was confirmed using primers XcpM1 (5′-ACGCGCTACCAAAAGGCAAAGAG-3′) and XcpM2 (5′-GATCTGCGGTTGTCCTGAAGATTGG-3′) in conventional PCR assays (Sulzinski et al., 1996).

#### Experiment 2. Direct Interaction of *B. methylotrophicus* OB9 With *Salmonella enterica* Newport SL1 or With *X. campestris* B07.007 on Leaf Surfaces

Tomato and lettuce leaves were submerged in 100 mL (10^6^ cells mL^–1^) of *B. methylotrophicus* OB9 in a petri plate for 30 s and then left for 2 h. Aliquots (100 μL) of each of *Salmonella* and *Xanthomonas* strains (10^6^ cells mL^–1^) were spread on the leaves, and incubated in petri plates at 22°C. Inoculums of *Salmonella* or *Xanthomonas* strains were also spread on non-inoculated tomato or lettuce leaves and served as experimental controls. After 72 h of incubation, recovery of bacterial cells from the leaves was determined by tissue maceration and cell count determination as described above.

### Statistical Analysis

In the co-culture experiment, there were three replicates per target organism and treatment, and the assays were repeated twice. In the tissue culture experiments (Experiment 1 and Experiment 2), each experimental unit consisted of one leaf per petri plate (one replicate). There were five replicates per treatment, and the entire experiment was repeated twice. Data of bacterial cell counts in co-culture and in tissue-culture experiments (Experiments 1 and 2) were analyzed by One-way ANOVA using the JMP 13.0 software. All experimental data were tested for statistical significance using Tukey’s HSD (*P* ≤ 0.05).

## Results

### Bacterial Diversity of Crude Oil-Inhabiting Bacteria

**FIGURE 1:**
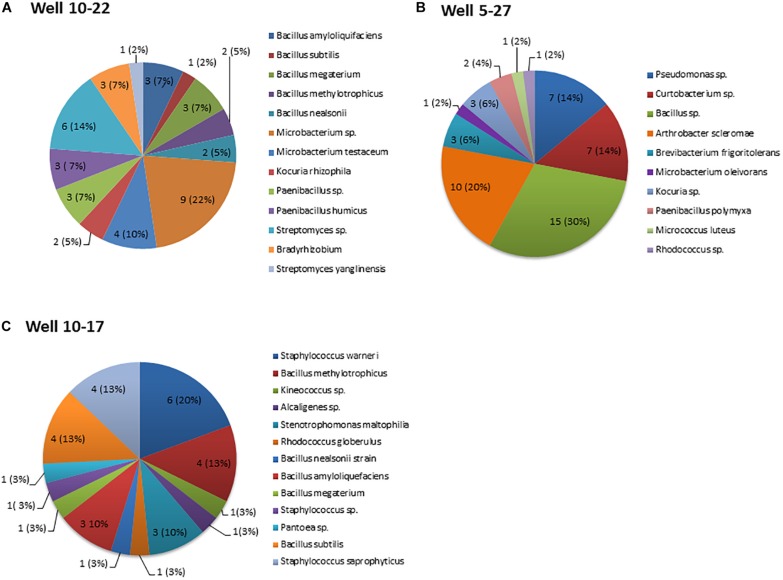
Distribution of bacterial genera recovered from the three oil wells. The numbers in each pie chart represent the isolates of each genus. The numbers in brackets represent the abundance of bacterial isolates of each genus relative to the total number of isolates recovered from each oil well. **(A)** Well 10–22, **(B)** Well 5-27, and **(C)** Well10-17. Data represent the average of three-replicate samples.

A total of 123 culturable bacteria, originating from crude oil samples were isolated from three oil batteries. A snapshot of the bacterial species in each oil battery, based on 16S rRNA gene sequences, indicate that the oil-inhabiting bacterial community is diverse (Figure 1) and composed predominantly of bacteria (the majority identified to the species level) from three phyla (Actinobacteria, Proteobacteria, and Firmicutes) along with 17 discrete genera that shared high homology with known sequences. The sequences of all strains have been deposited to GenBank under the following accession numbers (battery 10-22 MG924907-MG924915 and MG926585-MG926632, battery10-17 MH627942-MH627972, and battery 5-27 MG946770-MG946789, and MG951760-MG951768). The relative abundance of strains belonging to the Gram-positive *Bacillu*s genera (42, 39, and 32% from battery 10–17, 10–22, and 5–27, respectively) were the most common isolated across the three oil wells (Figure 1). A total of 42 strains recovered from oil battery 10–22 were distributed among six different genera with the highest relative abundance among isolates being *Bacillus* (11/42), *Microbacterium* (13/42) and *Streptomyces* (7/42) species (Figure 1A). Fifty isolates were recovered from Oil-battery 5–27 and belonged to 10 different genera, with *Bacillus* isolates again having the highest relative abundance (15/50). This battery also had isolates of *Arthrobacter* (10/50), *Pseudomonas* (7/50), *Curtobacterium* (7/50) and *Brevibacterium* (3/50) that were retrieved only from this oil well (Figure 1B). Lastly, there were 31 isolates representing seven different genera recovered from well 10–2717, with isolates of *Staphylococcus* (11/31), *Stenotrophomonas* (3/31), *Alcaligenes* (1/31), *Kineococcus* 1(31) and *Pantoea* (1/31) associated exclusively with this well (Figure 1C).

### Biosurfactant Production

The 34 isolates associated with *Bacillus, Streptomyces, Microbacterium, Micrococcus, Rhodococcus*, *Pseudomonas, Arthrobacter* and *Staphylococcus* genera retrieved from the three oil wells were identified as biosurfactant-producing bacteria using the oil–spreading and the drop collapse methods (Table 2). Isolates of *Bacillus* species showed the highest biosurfactant activities based on both tests. Strain OB9 isolated from oil well 10–22 and identified as *B. methylotrophicus* by whole genome sequencing (Jeukens et al., 2015) exhibited the highest biosurfactant activity followed by four strains of *B. amyloliquefaciens* (strains OB5, OB6, M30, and M09). Furthermore, these strains also tested positive for biosurfactant activity using the blood agar lysis method (a zone of 2.5 cm or greater), the CTAB method (a clearing zone of more than 1 mm), a significant grid using the microplate assay and in the emulsification assays (OD_600_ value of 1.0) (Supplementary Figure S2).

### Antimicrobial Activity of *B. methylotrophicus* OB9

**FIGURE 2:**
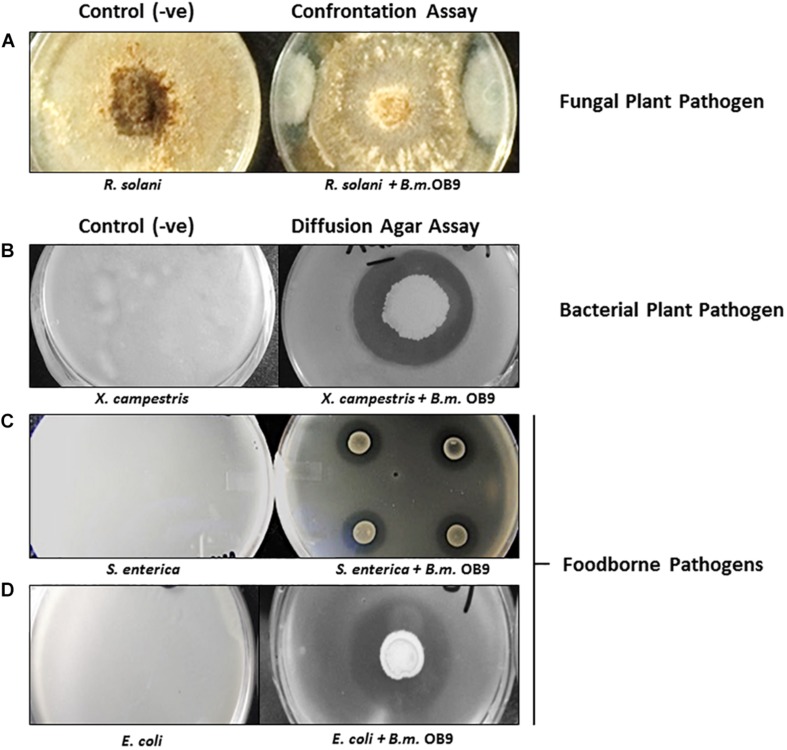
The antimicrobial activities of *B. methylotrophicus* OB9 (*B.m*.OB9) against plant and foodborne pathogens. **(A)** Confrontation co-culture assay with the fungal plant pathogen, *Rhizoctonia solani*. **(B)** Agar diffusion assays with *Xanthomonas campestris* B07.007. **(C)** *Salmonella enterica* Newport SL1. **(D)** *Escherichia coli* E14-6. The (−ve) series denotes confluent growth of each organism on the control plates, while in the (+ve) series the same organisms are challenged with *B. methylotrophicus* OB9 (as noted by the zone of clearing around *B. methylotrophicus* OB9).

*Bacillus methylotrophicus* OB9 was effective against all test bacteria and fungi with varying degrees of antimicrobial activity (Supplementary Table S2 and Figure 2). More specifically, live cultures of *B. methylotrophicus* OB9 inhibited the growth of 23 *Salmonella* serovars (environmental, clinical and food-borne isolates), 4 strains of *E. coli*, 10 environmentally isolated *Enterobacteiaceae* strains and a *X. campestris* pv. Vitians isolate (Supplementary Table S2 and Figures 2B–D) in agar diffusion assays, as visualized by a clear zone around the *B. methylotrophicus* OB9 colony that developed over 24–48 h. The diameter of the inhibition zone ranged from 3 to 6 mm and with some *E. coli* and *Salmonella* serovars a clear zone with double rings was observed (Supplementary Table S2), indicating the potency of one or more antimicrobials. The greatest zones of inhibition (6 mm in diameter) in response to *B. methylotrophicus* OB9 were observed in trials with *X. campestris* pv. Vitians and *Salmonella* serovar I:Rough-O:e,h:e,n (Figures 2B,C). Other *Salmonella* serovars (I:4,5,12:b:-, I:Rough-O:-:e,n,x, Hartford, and Heidelberg) were also highly sensitive to *B. methylotrophicus* OB9, exhibiting clearing zones of 5 mm. Interestingly, the biofilm-forming phenotypes (i.e., rdar-denoting the presence of the extracellular polysaccharide, cellulose, and curli fimbrae; saw – denoting the absence of cellulose and curli; Supplementary Table S1) of the *Salmonella* strains did not affect the ability of *B. methylotrophicus* OB9 to elicit zones of clearing. Even all of the control *S. enterica* Typhimurium strains that express both cellulose and curli (UMR1), only cellulose (MAE14), only curli fimbriae (MAE299) or neither component (MAE775) had a measurable zone of clearing around the *B. methylotrophicus* OB9 colony on the plates. These results suggest that the antimicrobial activities of *B. methylotrophicus* OB9 are not significantly affected by the biofilm barrier that bacterial cells use to exclude other antimicrobial agents (including disinfectants and antibiotics). The widespread effectiveness of *B. methylotrophicus* OB9 is further exemplified by its ability to also inhibit the growth of plant pathogenic fungi *Rhizoctonia solani* and *Botrytis cinerea*, as observed in the dual confrontation assays (Supplementary Table S2 and Figure 2A).

### Fractionation of Biosurfactant From *B. methylotrophicus* OB9

**FIGURE 3:**
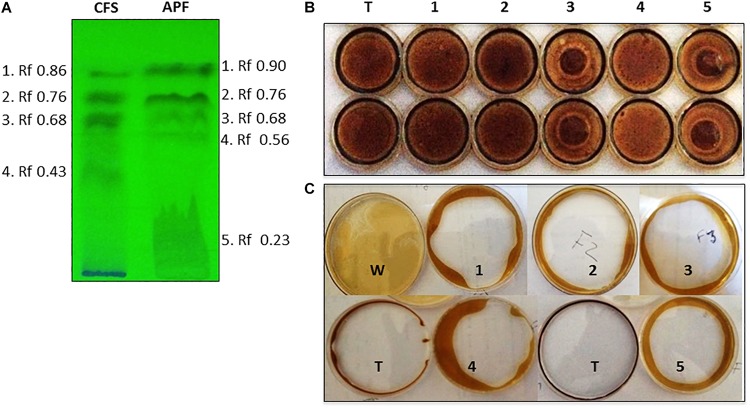
Thin layer chromatography (TLC) separation and biosurfactant assessment of CFS and APF extracts from *B. methylotrophicus* OB9. **(A)** TLC plate exposed to UV light with fraction numbers and Rf values denoted for crude (CFS) and acid precipitate (APF) preparations. The biosurfactant properties of TLC-isolated APF fractions of *B. methylotrophicus* OB9 were analyzed by the drop collapse assay **(B)** with APF fractions 1-5 and Triton X-100 (T) as a control and the oil spreading assay **(C)** with APF fractions 1-5, water (W) and Triton X-100 (T).

Biosurfactants were partially purified from culture filtrates of *B. methylotrophicus* OB9 grown in BHM broth enriched with oil. The purification procedure consisted of HCl precipitation, concentration of the precipitate by freeze-drying, dissolution in methanol-chloroform and analysis by TLC (Figure 3A). Fractionation of cell free supernatant (CFS) yielded four fractions (1–4), while the acid precipitate fraction (APF) separated into five parts. Between the CFS and APF samples, fractions 2 and 3 from the respective samples had identical Rf values (0.68 and 0.23) (Figure 3A).

### Biosurfactant and Antimicrobial Activity of the Acid Precipitate Fraction (APF) of *B. methylotrophicus* Strain OB9

**FIGURE 4:**
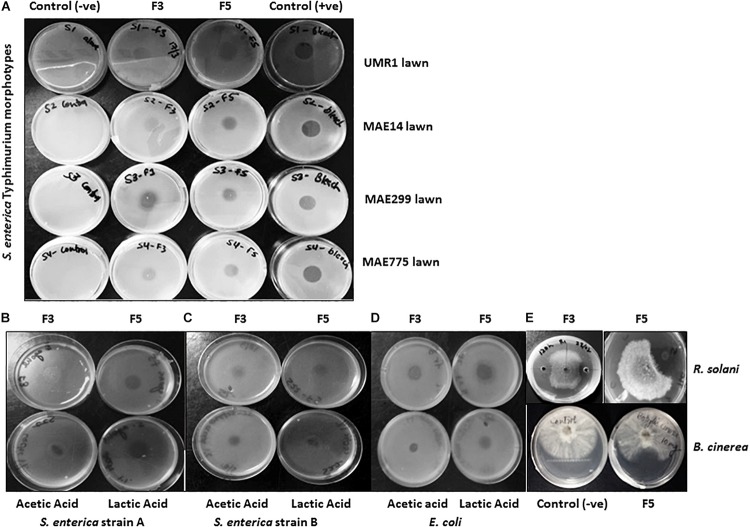
Antimicrobial assessment of APF extracts from *B. methylotrophicus* OB9. **(A)** Antimicrobial activity of APF fractions F3 and F5 with the agar diffusion assay against four biofilm morphotypes of *S. enterica Typhimurium.* From left to right: column 1 contains negative controls (–ve) for growth of each of the four strains (rows 1–4 are UMR1, MAE14, MAE299 and MAE775, respectively); columns 2–4 contain the same strains, but assayed against fraction 3 (F3), fraction 5 (F5) or bleach as positive control (+ve), respectively. Additional diffusion assays of F3, F5, 1% (v/v) acetic acid, and 1% (v/v) lactic acid against *S. enterica* Agona PARC#5, *S. enterica* serovar I:Rough-O:e,h:e,n and *E. coli* E14-6 are depicted in **(B–D)**, respectively. **(E)** The antagonistic effect of F3 and F5 against *Rhizoctonia solani* is depicted in the top two panels, while the bottom two panels display negative control growth (−) and APF fraction 5 (F5) assayed against *Botrytis cinerea*. Antimicrobial assessment of APF was conducted three times.

Only APF fractions (1–5) were assayed for biosurfactant and antimicrobial activities due to their improved purity and concentration relative to the CFS samples. All of the 5 TLC purified fractions of APF showed biosurfactant activity in the drop-collapse and oil-spreading assays (Figures 3B,C). However, only fractions 3 and 5 had strong antimicrobial activities in diffusion agar plates against 13 test bacteria. This test panel included eight *Salmonella* serovars originating from the environment, human fecal matter, or contaminated fresh produce, as well as strains of *E. coli* and *X. campestris* (a plant pathogenic bacterium) (Table 3 and Figures 4A–D). Additionally, the APF fractions were moderately effective against two fungal plant pathogens (Table 3 and Figure 4E); thereby indicating that these fractions have widespread microbial bioactivity. Furthermore, the size of the inhibition zones produced by the APF fractions (especially fraction 5) was comparable to that produced by the positive controls, bleach (Figure 4A) or common organic acids (lactic and acetic acid controls; Figures 4B–D) used as disinfectants and antimicrobials. Interestingly, the crude APF fraction (i.e., prior to TLC separation into fractions 3 and 5) exhibited only weak antimicrobial activity against the 13 bacteria and only one of the fungi (Table 3). Thus, isolation and partial enrichment of these fractions has increased their relative concentrations and/or efficacy.

**TABLE 3:** Microbial activity of the *B. methylotrophicus* OB9 fractions, organic acids and bleach against various pathogenic bacteria and fungal strains.

No	Stain ID	Identity	Live cells	APF	TLC fractions		Organic acids^a^				
					F3	F5	AA	LA	FA	CA	Bleach 1.3%
1	UMR1	*Salmonella enterica* Typhimurium.	+++	+	++	+++	++	+	+++	+	++
2	MAE14	*S. enterica* Typhimurium	+	+	++	++	++	++	+++	+	++
3	MAE299	*S. enterica* Typhimurium	+	+	+++	++	++	+	+++	+	++
4	MAE775	*S. enterica* Typhimurium	++	+	++	++	+++	+	+++	+	++
5	SCS2	*S. enterica* I:Rough-O:e,h:e,n.	++++	+	++	++	++	+	+++	+	++
6	*PARC#5*	*S. enterica* Agona	++	+	+	++	++	+	+++	+	++
7	SL1	*S. enterica* Newport	++	+	++	++	++	+	+++	+	++
8	SL2	*S. enterica* Hartford	++	+	++	++	++	+	+++	+	++
9	B07.007	*Xanthomonas campestris*	++++	+	++	+++	+++	++	+++	++	++
10	E3-6	*Escherichia coli*	+	+	++	++	++	+	+++	+	++
11	E10-6	*E. coli*	++	+	++	++	++	+	+++	+	++
12	E14-6	*E. coli*	+++^b^	+	++	++	++	+	+++	+	++
13	E15-6	*E. coli*	+	+	++	++	++	+	+++	+	++
14	AG3 (114)	*Rhizoctonia solani*	+++	+	+	++	++	+	+++	+	++
15	AG1-1A (ROS-2A)	*R. solani*	+	*-*	+	+	++	+	+++	+	++
16	F014	*Botrytis cinerea*	+	*-*	+	+	++	+	+++	+	++

*^*a*^Various organic acids (1%) tested as controls: AA, acetic acid; LA, lactic acid; FA, formic acid; CA, citric acid. ^*b*^Double ring of clearing zone. (+) clearing zone of 3 mm; (++) clearing zone of 4 mm; clearing zone of 5 mm (+++); clearing zone of 6 mm (++++).*

### Suppression of *Salmonella* and *Xanthomonas* Growth During Co-culture With *B. methylotrophicus* OB9

**FIGURE 5:**
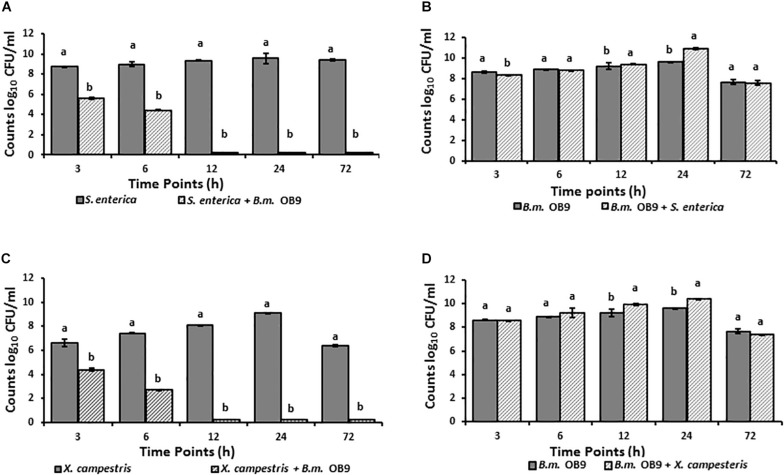
Suppression of *Salmonella* and *Xanthomonas* growth in co-cultures with *B. methylotrophicus* OB9. Temporal microbial counts of *S. enterica* Newport (SL1) **(A)** and *B. methylotrophicus* (*B.m*.OB9) **(B)** in single culture and co-cultures over 72 h. Temporal microbial counts of *Xanthomonas* **(C)** and *B. methylotrophicus* OB9 **(D)** in single culture and co-culture over 72 h.

*Salmonella* cell numbers in liquid co-cultures with *B. methylotrophicus* OB9 significantly (*P* < 0.05) decreased at 3 h (31-fold) and 6 h (45-fold) after incubation compared to growth controls (Figure 5A). Similarly, *X. campestris* was recovered 3 and 6 h after incubation in co-cultures, with significant decreases of 22 and 47-fold in cell number, respectively (Figure 5C). Furthermore, neither *S. enterica* Newport or *X. campestris* were retrieved from co-cultures after 12 h or more of incubation (Figures 5A,C), while *B. methylotrophicus* OB9 continued to grow at a comparable rate to cultures of *B. methylotrophicus* OB9 alone (Figures 5B,D). To rule out the possibility of nutrient competition as a source of the antagonistic behavior, media was replaced every 10 h, but this did not affect results.

### Recolonization, Internalization, and Detection of Bacterial Isolates in Plant Tissues

**FIGURE 6:**
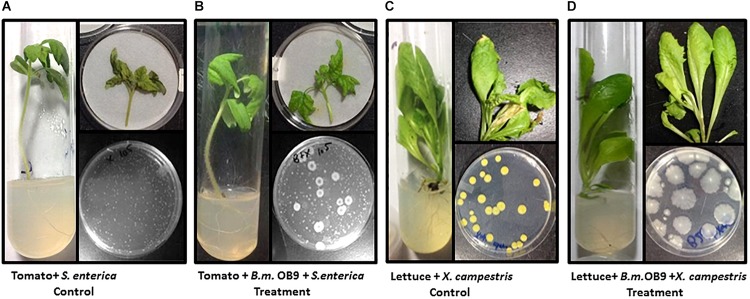
Suppression of *Salmonella* and *Xanthomonas* growth in bacterized plants with *B*. *methylotrophicus* OB9. **(A)** Control treatment (tomato + *S. enterica* Newport SL1) displays a tomato seedling in tissue culture infected with *S. enterica* Newport SL1. Recovery of *S. enterica* Newport SL1 from an infected detached leaf representative of CFU/gram tissue is depicted on the agar plate. **(B)** Treatment (Tomato + *B.m* OB9 + *S. enterica*)-consisting of a tomato seedling in tissue culture bacterized with *Bacillus methylotrophicus* OB9 and infected with *S. enterica* Newport SL1. Recovery of OB9 and SL1 cells expressed as CFU/gram tissue as depicted on the agar plate. **(C)** Control treatment (Lettuce + *X. campestris*) showing a lettuce seedling in tissue culture infected with *X. campestris* B07.007. Recovery of *X. campestris* cells on culture medium from infected leaves indicative of the CFU/gram tissue. **(D)** Treatment (lettuce + *B.m.*OB9 + *X. campestris)* of a lettuce seedling in tissue culture bacterized with *B. methylotrophicus* OB9 and infected with *X. campestris* B07.007. Recovery of *B. methylotrophicus* OB9 but not *X. campestris* B07.007 on culture medium from detached bacterized leaves treated and infected with *X. campestris* B07.007 as depicted on the agar plate and representative of the CFU/gram tissue.

The surface-sterilization method combined with the imprint technique ensured that the endophytic colonization numbers reflect only the cells within the interior of the plant tissues (i.e., bacterized), as non-colonized plants did not yield culturable bacterial colonies (data not shown). Subsequent isolation and quantification of *B. methylotrophicus* (OB9) following surface sterilization demonstrated that the strain can travel from the roots to the stems and leaves and develop sustained endophytic populations in plant tissues of tomato (log_10_7) and lettuce (log_10_8) grown under gnotobiotic conditions even after the plants were challenged with *Salmonella* and *Xanthomonas* strains (Figures 5, 6).

### Suppression of *Salmonella* and *Xanthomonas* Growth in Bacterized Plants With *B. methylotrophicus* OB9

**FIGURE 7:**
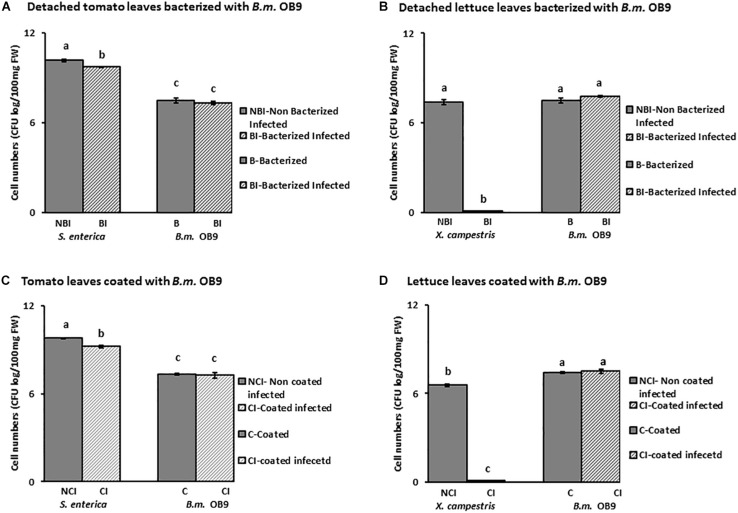
Quantification of cell numbers of *B. methylotrophicus* OB9, *S. enterica* Newport SL1 and *X. campestris* B07.007 recovered from leaf tissues. Leaf tissues from bacterized with *B. methylotrophicus* (*B.m*.OB9) and challenged with *S. enterica* Newport SLI and *X. campestris* are represented in **(A,B)**, respectively. Tomato and lettuce leaf samples that were coated with OB9 and infected with *S. enterica* Newport SLI and *X. campestris* are denoted by **(C,D)**, respectively. (BI) and (CI) samples represent *B. methylotrophicus* OB9 bacterized (BI) or coated (CI) tissues that are infected with either *S. enterica* Newport SLI or *X. campestris* (NBI) and (NCI) treatments represent non-bacterized and non-coated but infected with *S. enterica* Newport SLI or *X. campestris* controls, respectively. Data represent the average of 5 replicates per treatment.

Leaves of *B. methylotrophicus* (OB9) bacterized tomato and lettuce plants challenged with *Salmonella* and *Xanthomonas* appeared healthy compared to challenged non-bacterized plants (Figure 6). There was a significant (*P* < 0.05) reduction of *Salmonella* cells (44% or 4.4 fold -decrease) recovered from bacterized tomato leaves after 72 h (Figures 6, 7A) compared to challenged non-bacterized leaves. In the case of *Xanthomonas*, there was no growth as reflected by complete absence of cells from bacterized lettuce tissues after 72 h (Figures 6, 7B). Amplification of genomic DNA extracted from bacterized leaf samples using designed *B. methylotrophicus* OB9 specific primers gave the expected band size of 565 bp in leaf and root tissue samples (data not shown). Similarly, amplification of *Salmonella* from bacterized plants gave the expected band size of 429 bp, so the presence/absence of these bacteria was confirmed by this secondary method (data not shown). PCR amplification of *Xanthomonas* DNA was not successful using the XcpM 1/XcpM2 primers so this organism could not be detected in coated and challenged lettuce leaves by this secondary method.

In a follow-up experiment, where tomato and lettuce leaves were coated with *B. methylotrophicus* OB9 (as opposed to bacterized) and challenged with *Salmonella* and *Xanthomonas*, similar trends were noted to the bacterized experiment. Recovery of *Salmonella* cell-numbers from these leaves was significantly decreased by 54% (5.4-fold decrease) compared to leaves treated with *Salmonella* alone (Figures 7B,C). As in the bacterized experiment, *Xanthomonas* was not recovered in the *B. methylotrophicus* OB9 coating experiments either (Figure 7D).

## Discussion

Bacterial communities in crude oil samples from oil wells in the Chauvin area (Alberta, Canada) were assessed for the first time in this study. The bacterial distribution in these oil wells revealed that each well had distinct bacterial community structure, however, strains belonging to the genus *Bacillus* were common and some of the most prevalent among the three oil wells. One may argue that the diversity of bacterial communities could be the result of introducing exogenous species from injection of water into the wells, a common procedure used in crude oil extraction (Magot et al., 2000). However, the extreme environment of oil wells (i.e., high temperature, salinity and pressure) is proposed to favor the growth of indigenous bacterial communities and depress foreign species (Wang et al., 2014). Furthermore, our results are in agreement with previous reports on microbial diversity in other oil well reserves (Lenchi et al., 2013; Xiao et al., 2016). The biosurfactant-producing bacteria identified in this study (*Bacillus, Streptomyces*, *Microbacterium Pseudomonas, Arthrobacter, Rhodococcus* and *Micrococcus*) belong to genera that have also been identified in a wide range of studies as crude oil degraders and biosurfactant producers (Wu et al., 2008; Brooijmans et al., 2009; Korenblum et al., 2012; Obi et al., 2016; Parthipan et al., 2017; Xu et al., 2018). In follow-up studies, the oil-spreading and drop collapse assays allowed for confirmation and initial ranking of our isolated biosurfactant producers (Santos et al., 2016). The battery of subsequent assays clearly demonstrated that *B. methylotrophicus* OB9 had an elevated ability to produce biosurfactants. These results parallel other reports on crude oil-dwelling strains of *B. methylotrophicus* as efficient biosurfactant producers (Chandankere et al., 2013; Jemil et al., 2016; Chaprão et al., 2018).

One of the goals of this study was to identify newly characterized microbes with prophylactic abilities against food-borne and plant pathogens. Food-borne bacteria, such as *Salmonella* strains, are known to exist in biofilms, which consist of the bacteria embedded in an extracellular matrix that enhances adherence and persistence to/on abiotic and biotic surfaces, including fresh produce and other surfaces along the food supply chain (Stepanoviæ et al., 2004). The biofilm matrix is self-produced by the embedded bacteria and consists predominantly of a mixture of protein, carbohydrate and nucleic acid material. For example, many *Salmonella* and *E. coli* strains can produce curli fimbriae and the exopolysaccharide, cellulose, that are involved in surface adhesion, cell aggregation and persistence of these bacteria in various environments (Zogaj et al., 2001). Loss or disruption of any of these components leads to distinct colony morphotypes and decreased persistence/survival outside human hosts or on plants, such as fruits and vegetables (Milanov et al., 2015). In this study, the evaluation of the antimicrobial activity of *B. methylotrophicus* OB9 in agar-diffusion assays was evidenced against our complete panel of control *Salmonella enterica* Typhimurium strains displaying various biofilm expression morphotypes (UMR1, MAE14, MAE299 and MAE775); thereby indicating that the antagonistic activities of *B. methylotrophicus* OB9 are not circumvented by the biofilm barrier and that this strain holds promise as an alternative biocontrol strategy to that of antibiotics and disinfectants. Furthermore, the antagonistic capability of *B. methylotrophicus* OB9 was also noted against 17 other *Salmonella* serovars, including top clinical serovars Typhimurium, Heidelberg, Newport, Infantis, Thompson and Braenderup, as well as environmental isolates. The activity of *B. methylotrophicus* OB9 also proved effective on a broader scale, where it affected 4 strains of *E. coli*, with the highest activity against *E. coli* E10-6, and a virulent strain of *X. campestris*, the causal agent of BLS on lettuce. *B. methylotrophicus* OB9 was antagonistic against the growth of fungal varieties as well, especially *R*. *solani*. Therefore, *B. methylotrophicus* OB9 is a strong candidate as a general biological control agent against both food-borne and plant pathogens.

To gain a better understanding of the fractional component(s) of *B. methylotrophicus* OB9 responsible for antimicrobial activity, standard cell partitioning methods [HCl precipitation (Alajlani et al., 2016)] were used and the resultant fractions tested for their biosurfactant and antimicrobial activity against our panel of food-borne and plant pathogens. Microbial biosurfactants are a broad group that may consist of lipids, glycolipids, lipopeptides and/or polysaccharide-protein complexes (Roy, 2017; Varjani and Upasani, 2017; Akbari et al., 2018). More specifically, *Bacillus* species are known to produce a multitude of secondary active metabolites, such as antibiotics and broad-spectrum lipopeptide biosurfactants (e.g., surfactin, iturin, and fengycin families) that display antagonistic activities that make them ideal biological control agents (McSpadden Gardener, 2004). Toward this end, we previously characterized the cyclic lipopeptides, including surfactin, iturin and mycobacillin, produced by *B. methylotrophicus* strain B26 associated with the switchgrass bioenergy crop (Gagne-Bourgue et al., 2013). With respect to antifungals, lipopeptides are extensively used in feed, plant defense and food preservation (Cawoy et al., 2015; Meena and Kanwar, 2015), but the use of other secondary active metabolites against bacteria also holds promise. While biochemical characterization of specific biosurfactants is merited, the immediate goal of this study was to identify the sub-cellular purified fractions with the greatest antagonistic activity, which happened to include biosurfactants. Specifically, the TLC-separated fractions 3 and 5 were effective in reducing surface tension compared to Triton X-100 (i.e., indicative of biosurfactants) and displayed broad-spectrum inhibitory effects equal in intensity to acids and bleach. These results are significant given that the presence of curli fimbriae, cellulose and a biofilm-associated protein (BapA) in *Salmonella enetrica* serovar Enteriditis biofilms conferred resistance to these biocontrol agents (Gibson et al., 2006). Thus, it is also noteworthy that the activity of fractions 3 and 5 were antagonistic against our entire panel of *Salmonella* biofilm controls and 17 other serovars, as well as the *E. coli, X. campestris* and fungal strains. Thus, these fractions appear to account for all of the activity noted in the *B. methylotrophicus* OB9 live cell assays and in some cases was more potent than the live cultures, possibly due to the purity and/or elevated concentrations of the antimicrobial agent(s) in these fractions.

A number of studies have demonstrated the ability of human pathogenic bacteria to colonize not only the surface of the host plant, but also the interior; thereby raising concerns of contracting food-borne illness from vegetables (Fletcher et al., 2013). Recently reported outbreaks of *E. coli* O157:H7 on fresh lettuce and spinach, as well as Salmonellosis from hot peppers underscore these concerns (Fletcher et al., 2013). High numbers of *S. enterica* Newport SL1 have also been noted to adhere to alfalfa sprouts, which was directly related to their ability to produce aggregate curli fimbriae (Barak et al., 2002). In this study, *S. enterica* Newport SL1 sustained reasonable population numbers on tomato leaves, which is in agreement with a large body of evidence that many *Salmonella* serovars, including Newport, can attach, form biofilms and grow/survive on diverse plant hosts (Schikora et al., 2012); prompting their classification as generalist microbes.

The bacterization of tomato seedlings by *B. methylotrophicus* OB9 led to colonization and internalization of the tissues of tomato seedlings. The sustained population in leaf tissues under gnotobiotic conditions over time suggests that this bacterium is an endophyte, given that bacterized plants showed no symptoms of stress. Bacterial endophytes can confer tolerance or resistance to the host plant from biotic and abiotic stresses by releasing antimicrobial compounds, producing siderophores, competing for space and nutrients, and modulating the plant resistance response (Santoyo et al., 2016; McNees et al., 2019). Given the success of *B. methylotrophicus* OB9 (or fractions thereof) as antimicrobial agents in our screening assays, we wanted to further explore the potential protective behavior of this endophyte *in planta*. As proof-of-concept we evaluated the ability of *B. methylotrophicus* OB9 to protect vegetables from contamination by *S. enterica* Newport SL1 (human pathogen) and from *X. campestris* B0.007 (a pathogen of tomato and lettuce) under gnotobiotic systems. The number of *S. enterica* Newport SL1 was significantly reduced in either *B. methylotrophicus* OB9 bacterized or coated tomato leaves. Likewise, detached lettuce leaves of bacterized plants or coated leaves with *B. methylotrophicus* OB9 had drastic effects on the persistence of this bacterial pathogen, as it was completely eliminated from the leaves of lettuce in both cases. The results of the cell-fractionation experiments suggest that the antimicrobial nature of the results *in planta* may be directly due to the release of antimicrobial compounds, such as biosurfactants, but the possibility of indirect effects from increasing plant defense mechanisms or a combination of both these possibilities cannot be ruled out (Khare et al., 2018). Further evidence of how *B. methylotrophicus* OB9 protects against *Salmonella* and *Xanthomonas* may help to delineate these possibilities.

In summary, this study successfully isolated and characterized 123 strains of crude oil-dwelling bacteria and comprehensively assessed the antimicrobial activity of an efficient biosurfactant-producing *B. methylotrophicus* OB9 strain. The inhibition spectrum of this strain against diverse human and plant pathogens makes it attractive for agricultural and food safety applications. This possibility is underscored by the fact that biofilm morphotypes of *Salmonella* were equally susceptible to *B. methylotrophicus* OB9 (and specific fractions thereof), thus indicating that the traditional biofilm barrier for exclusion of antibiotics and disinfectants is not effective against *B. methylotrophicus* OB9. Further purification and characterization of the biosurfactants produced by this strain is warranted for future use of the bacteria or other antimicrobial metabolites as biological controls against food-borne pathogens and bacterial pathogens of leafy vegetables or toward the development of new drugs for problematic pathogenic bacteria.

## Data Availability Statement

The datasets generated for this study can be found in the GenBank (https://www.ncbi.nlm.nih.gov/nucleotide/) under the following accession numbers: battery 10-22 MG924907–MG924915 and MG926585–MG926632, battery10-17 MH627942–MH627972, and battery 5-27 MG946770–MG946789, and MG951760–MG951768.

## Author Contributions

SJ designed the experiments. MR conducted the experiments. SJ and MR interpreted the results, and drafted the manuscript. JW was consulted on results interpretation and drafting/revising the manuscript. All authors read and approved the final manuscript.

## Conflict of Interest

A patent has been filed by McGill University under the number 62/979,570. The authors declare that the research was conducted in the absence of any commercial or financial relationships that could be construed as a potential conflict of interest.

## Footnotes

1 http://www.ncbi.nlm.nih.gov/blast

## Supplementary Material

FIGURE S1 Location of oil wells, 10–22, 5–72, and 10–17 in Chauvin region.

FIGURE S2 Assays for Biosurfactant production. Examples of different physical assays are displayed for the **(A)** Oil spreading assay, **(B)** CTAB agar assay, **(C)** Emulsification capacity assay, **(D)** Blood agar plate and **(E)** Drop collapse assay, with left to right denoting negative, positive and four test samples (S1–S4). **(F)** Microplate assay; from left to right displaying negative, positive controls and a test sample (S1).

TABLE S1 Source of organisms used in the study.

TABLE S2 Antagonistic activity of OB9 cultures against bacteria and fungi.
