# Supplementary figures and images for: Isolation and Characterization of Biosurfactant-Producing Bacteria From Oil Well Batteries With Antimicrobial Activities Against Food-Borne and Plant Pathogens
Source: Front Microbiol. 2020 Feb 27;11:64. doi: 10.3389/fmicb.2020.00064 (PMC7093026; doi:10.3389/fmicb.2020.00064)

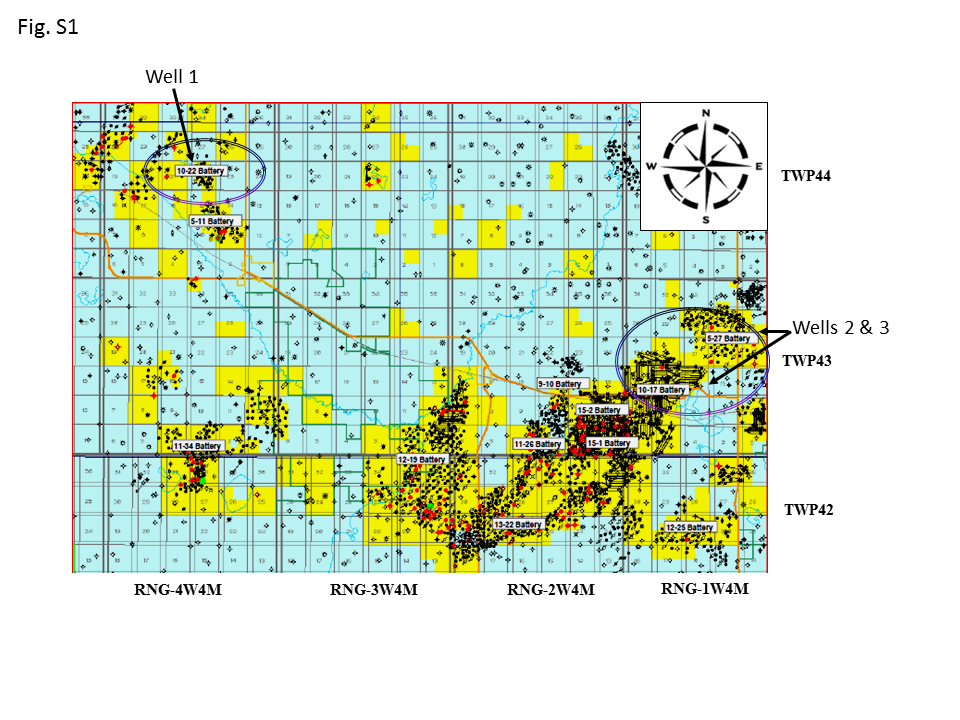

Supplement: FIGURE S1 — Location of oil wells, 10–22, 5–72, and 10–17 in Chauvin region. [file Image_1.TIF]

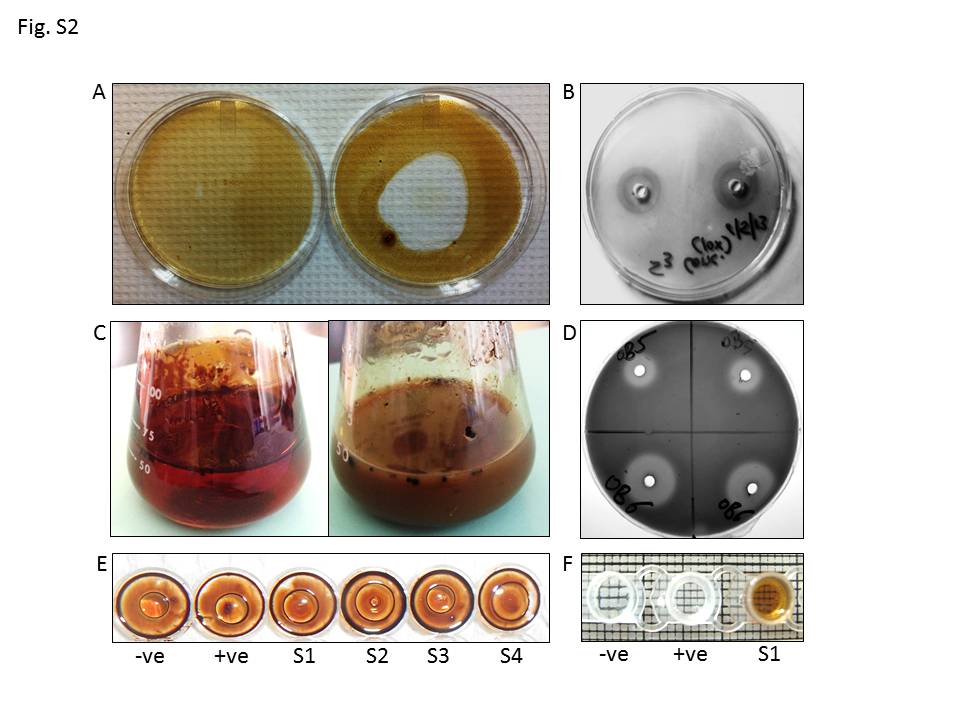

Supplement: FIGURE S2 — Assays for Biosurfactant production. Examples of different physical assays are displayed for the (A) Oil spreading assay, (B) CTAB agar assay, (C) Emulsification capacity assay, (D) Blood agar plate and (E) Drop collapse assay, with left to right denoting negative, positive and four test samples (S1–S4). (F) Microplate assay; from left to right displaying negative, positive controls and a test sample (S1). [file Image_2.TIF]
