# Supplementary material for: Isolation and Characterization of Biosurfactant-Producing Bacteria From Oil Well Batteries With Antimicrobial Activities Against Food-Borne and Plant Pathogens
Source: Front Microbiol. 2020 Feb 27;11:64. doi: 10.3389/fmicb.2020.00064 (PMC7093026; doi:10.3389/fmicb.2020.00064)
Supplement: TABLE S1 — Source of organisms used in the study. [file Table_1.DOCX]

**Table 1S**: Source of organisms used in the study.

| **Number** | | **Stain ID** | | | **Identity** | | | | **Biofilm formation**  **at 28°C** | | | | **Origin/morphotype** | | | **Source/provider** |
| --- | --- | --- | --- | --- | --- | --- | --- | --- | --- | --- | --- | --- | --- | --- | --- | --- |
| BACTERIA |  | |  |  | |  |  |  | |  |  |  | |  |  | |
| *Salmonella strains/morphotypes* | |  | | |  | | | |  | | | |  | | |  |
| 1 | | UMR1 | | | *Salmonella typhimurium* | | | | rdar* | | | | Control | | | 1 |
| 2 | | MAE14 | | | *S. typhimurium* | | | | pdar* | | | | Control | | | 1 |
| 3 | | MAE299 | | | *S. typhimurium* | | | | bdar* | | | | Control | | | 1 |
| 4 | | MAE775 | | | *S. typhimurium* | | | | baw* | | | | Control | | | 1 |
| 5 | | S1V1 | | | *Salmonella sp.* | | | | pdar | | | | Environment | | | 1 |
| 6 | | S3PP | | | *Salmonella sp.* | | | | saw | | | | Environment | | | 1 |
| 7 | | S13V2 | | | *Salmonella sp.* | | | | rdar | | | | Environment | | | 1 |
| 8 | | S2V3 | | | *Salmonella sp.* | | | | rdar & saw | | | | Environment | | | 1 |
| 9 | | S1V3 | | | *Salmonella sp.* | | | | saw | | | | Environment | | | 1 |
| 10 | | S4V2 | | | *Salmonella sp.* | | | | pdar & saw | | | | Environment | | | 1 |
| 11 | | S10V1 | | | *Salmonella sp.* | | | | pdar | | | | Environment | | | 1 |
| 12 | | S12PP | | | *Salmonella sp.* | | | | rdar | | | | Environment | | | 1 |
| 13 | | S18V2 | | | *Salmonella sp.* | | | | pdar & saw | | | | Environment | | | 1 |
| 14 | | S2V2 | | | *Salmonella sp.* | | | | Combination | | | | Environment | | | 1 |
| 15 | | SC01 | | | *Salmonella Serotype l:4,5,12:b:* | | | | rdar | | | | Environment | | | 1 |
| 16 | | SC16 | | | *Salmonella Serotype l:Rough-O::e,n,x* | | | | saw | | | | Environment | | | 1 |
| 17 | | SC04 | | | *Salmonella Serotype Braenderup* | | | | rdar | | | | Environment | | | 1 |
| 18 | | SCS7 | | | *Salmonella Serotype Typhimurium* | | | | saw | | | | Environment | | | 1 |
| 19 | | SC110 | | | *Salmonella Serotype I:RoughO:y:e,n,x* | | | | rdar | | | | Environment | | | 1 |
| 20 | | SC12 | | | *Salmonella Serotype l:Rough-O:e,h* | | | | rdar | | | | Environment | | | 1 |
| 21 | | SC18 | | | *Salmonella Serotype Hartford* | | | | rdar | | | | Environment | | | 1 |
| 22 | | SCS2 | | | *Salmonella Serotype:RoughO:e,h:e,* | | | | rdar | | | | Environment | | | 1 |
| 23 | | WTCR5 | | | *Salmonella Serotype l:6,7:r* | | | | rdar/saw | | | | Environment | | | 1 |
| 24 | | WTCR6 | | | *Salmonella Serotype Stanley* | | | | rdar/saw | | | | Environment | | | 1 |
| 25 | | WTCR9 | | | *Salmonella Serotype Infantis* | | | | rdar/saw | | | | Environment | | | 1 |
| 26 | | WTCR30 | | | *Salmonella Serotype- Schwarzengrund* | | | | rdar/saw | | | | Environment | | | 1 |
| 27 | | WTCR22 | | | *Salmonella Serotype Thompson* | | | | rdar/saw | | | | Environment | | | 1 |
| 28 | | WTC27 | | | *Salmonella serotype Heidleberg* | | | | saw | | | | Environment | | | 1 |
| 29 | | WTC28 | | | *Salmonella Serotype Monschaui* | | | | rdar/saw | | | | Environment | | | 1 |
| 30 | | WTCT4 | | | *Serotype Heidleberg* | | | | rdar/saw | | | | Environment | | | 1 |
| 31 | | *PARC#5* | | | *S. agona* | | | | rdar/saw | | | | Mung bean | | | 3 |
| 32 | | SL1 | | | *S. Newport* | | | | rdar | | | | Human gut | | | 2 |
| 33 | | SL2 | | | *S. Hartford* | | | | rdar | | | | Human gut | | | 2 |
| *Escherichia* species | |  | | |  | | | |  | | | |  | | |  |
| 34 | | E3-6 | | | *Escherichia coli* | | | | NA | | | | NA | | | 1 |
| 35 | | E10-6 | | | *E. coli* | | | | NA | | | | NA | | | 1 |
| 36 | | E14-6 | | | *E. coli* | | | | NA | | | | NA | | | 1 |
| 37 | | E15-6 | | | *E. coli* | | | | NA | | | | NA | | | 1 |
| *Xanthomonas* sp. | |  | | |  | | | |  | | | |  | | |  |
| 38 | | B07.007 | | | *Xanthomonas campestris pv. vitians* | | | | rdar | | | | Lettuce | | | 4 |
|  | |  | | |  | | | |  | | | |  | | |  |
| FUNGI | |  | | |  | | | |  | | | |  | | |  |
| *Rhizoctonia* sp. | |  | | |  | | | |  | | | |  | | |  |
| 39 | | AG3-114 | | | *R. solani* | | | | NA | | | | Potato | | | 5 |
| 40 | | AG1-1-ROS-2A4) | | | *R. solani* | | | | NA | | | | Soybean | | | 6 |
| *Botrytis* | |  | | |  | | | |  | | | |  | | |  |
| 41 | | F-014 | | | *Botrytis cinerea* | | | | NA | | | | Strawberry | | | 7 |

*Strains displaying rdar (red dry and rough) are cellulose and curli positive, strains displaying pdar (pink dry and rough) are cellulose positive, strains displaying bdar (brown dry and rough) are curli positive, and strains displaying saw (negative for both components) are negative for both components, NA –not applicable.

1. .J. Weadge, Wilfrid Laurier University, Ontario; U. Romling *et al.,* (2003). Int. J. Med. Microbiol. 293, 273-285. **2.** S. Bekal, The Laboratory of Public Health of Quebec (QPHL), Quebec. **3.** S. Orban, Agriculture and Agri-Food Canada (AAFC), BC. **4.** V. Toussaint, AAFC, St. Jean sur Richelieu, Québec. **5**. M. A. Cubeta , N.C. States University, NC, USA. **6**. P. Ceresini*,* UNESP Sao Paolo, Brazil. **7**. S. Jabaji, McGill University, Quebec.
