# Supplementary material for: Isolation and Characterization of Biosurfactant-Producing Bacteria From Oil Well Batteries With Antimicrobial Activities Against Food-Borne and Plant Pathogens
Source: Front Microbiol. 2020 Feb 27;11:64. doi: 10.3389/fmicb.2020.00064 (PMC7093026; doi:10.3389/fmicb.2020.00064)
Supplement: TABLE S2 — Antagonistic activity of OB9 cultures against bacteria and fungi. [file Table_2.DOCX]

**Table 2S**: Antagonistic activity of OB9 cultures against bacteria and fungi

| **S.NO** | **Stain ID** | **Identity** | **OB9**  **Live cells** |
| --- | --- | --- | --- |
| 1 | UMR1 | *Salmonella typhimurium* | +++ |
| 2 | MAE14 | *S. typhimurium* | + |
| 3 | MAE299 | *S. typhimurium* | + |
| 4 | MAE775 | *S. typhimurium* | ++ |
| 15 | SC01 | *Serotype l:4,5,12:b:* | +++* |
| 16 | SC16 | *Serotype l:Rough-O::e,n,x* | +++* |
| 17 | SC04 | *Serotype Braenderup* | ++ |
| 18 | SCS7 | *Serotype Typhimurium* | + |
| 19 | SC110 | *Serotype I:RoughO:y:e,n,x* | ++ |
| 20 | SC12 | *Serotype l:Rough-O:e,h* | + |
| 21 | SC18 | *Serotype Hartford* | +++* |
| 22 | SCS2 | *Serotype:RoughO:e,h:e,* | ++++ |
| 23 | WTCR5 | *Serotype l:6,7:r* | + |
| 24 | WTCR6 | *Serotype Stanley* | + |
| 25 | WTCR9 | *Serotype InfaNtis* | ++ |
| 26 | WTCR30 | *Serotype- Schwarzengrund* | + |
| 27 | WTCR22 | *Serotype Thompson* | ++ |
| 28 | WTC27 | *serotype Heidleberg* | +++* |
| 29 | WTC28 | *Serotype Monschaui* | + |
| 30 | WTCT4 | *Serotype Heidleberg* | ++ |
| 31 | S. agona | *PARC#5 S. agona* | ++ |
| 32 | SL1 | *S. Newport* | ++ |
| 33 | SL2 | *S. Hartford* | ++ |
| 34 | B07.007 | *Xanthomonas hortorum* | ++++ |
| 35 | E3-6 | *Escherichia coli* | + |
| 36 | E10-6 | *E.coli* | ++ |
| 37 | E14-6 | *E. coli* | +++* |
| 38 | E15-6 | *E. coli* | + |
| 39 | AG3-114 | *Rhizoctonia solani* | +++ |
| 40 | AG1-1(ROS-2A4) | *R, solani* | + |
| 41 | F014 | *Botrytis cinerea* | + |

Clearing zone of and above 3mm is considered as positive; + clearing zone of 3mm, ++ clearing zone of 4mm, +++ 5mm , ++++ 6mm.

** Double ring of clearing zone.
